# Impact of a competency based curriculum on quality improvement among internal medicine residents

**DOI:** 10.1186/s12909-014-0252-7

**Published:** 2014-11-28

**Authors:** Mark C Fok, Roger Y Wong

**Affiliations:** Division of Geriatric Medicine, Department of Medicine, University of British Columbia, 7th Floor, 2775 Laurel Street, Vancouver, British Columbia V5Z 1M9 Canada; Dean’s Office, Postgraduate Medical Education, Faculty of Medicine, University of British Columbia, 11th Floor, 2775 Laurel Street, Vancouver, British Columbia V5Z 1M9 Canada

## Abstract

**Background:**

Teaching quality improvement (QI) principles during residency is an important component of promoting patient safety and improving quality of care. The literature on QI curricula for internal medicine residents is limited. We sought to evaluate the impact of a competency based curriculum on QI among internal medicine residents.

**Methods:**

This was a prospective, cohort study over four years (2007–2011) using pre-post curriculum comparison design in an internal medicine residency program in Canada. Overall 175 post-graduate year one internal medicine residents participated. A two-phase, competency based curriculum on QI was developed with didactic workshops and longitudinal, team-based QI projects. The main outcome measures included self-assessment, objective assessment using the Quality Improvement Knowledge Assessment Tool (QIKAT) scores to assess QI knowledge, and performance-based assessment via presentation of longitudinal QI projects.

**Results:**

Overall 175 residents participated, with a response rate of 160/175 (91%) post-curriculum and 114/175 (65%) after conducting their longitudinal QI project. Residents’ self-reported confidence in making changes to improve health increased and was sustained at twelve months post-curriculum. Self-assessment scores of QI skills improved significantly from pre-curriculum (53.4 to 69.2 percent post-curriculum [p-value 0.002]) and scores were sustained at twelve months after conducting their longitudinal QI projects (53.4 to 72.2 percent [p-value 0.005]). Objective scores using the QIKAT increased post-curriculum from 8.3 to 10.1 out of 15 (p-value for difference <0.001) and this change was sustained at twelve months post-project with average individual scores of 10.7 out of 15 (p-value for difference from pre-curriculum <0.001). Performance-based assessment occurred via presentation of all projects at the annual QI Project Podium Presentation Day.

**Conclusion:**

The competency based curriculum on QI improved residents’ QI knowledge and skills during residency training. Importantly, residents perceived that their QI knowledge improved after the curriculum and this also correlated to improved QIKAT scores. Experiential QI project work appeared to contribute to sustaining QI knowledge at twelve months.

## Background

Over the past decade, much effort has been invested to improve health by advancing the quality of health care with an emphasis of promoting patient safety and reducing medical error [1,2]. Accordingly, many training programs on quality improvement (QI) have been developed for practitioners in health care [3]. Most of these programs are constructed for continuous professional development (CPD) purposes aimed at individuals who have completed their clinical training, and relatively few are designed specifically for medical residents. Numerous barriers exist to implementing these CPD based QI training programs in residency training programs, including a lack of dedicated time in the core residency curriculum, limited faculty who have the expertise and/or interest in the topic, and a paucity of infrastructural support and financial resources.

A systematic review of residents’ engagement in quality improvement found that the role and participation of residents in a clinical QI initiative varied widely [4]. Few studies described the educational impact of residents’ participation in QI and even fewer studies identified specific improvement in patient health outcomes. More recent studies have focused on the development of core residency-specific QI curricula [5–8]. However, many programs are delivered over a short span of time (ranging from one day to one month elective blocks), thus creating the uncertainly of whether any short-term knowledge gains are sustained. Also, while theoretical constructs are taught, there is no or minimal component of clinical applicability. To overcome the latter concern, a practice-based QI elective rotation was recently offered to internal medicine residents, with promising results [9]. Specifically, residents who completed a QI project demonstrated superior knowledge retention of QI skills on objective testing when compared to non-completers. We were therefore interested in developing and evaluating the impact of a novel competency based curriculum on QI as measured by self-assessment of QI attitudes and objective assessment of QI knowledge. The curriculum was tailored to the needs of internal medicine residents, with longitudinal content delivery and with a team-based project component.

A competency is an observable ability of a health professional, integrating multiple components such as knowledge, skills, values and attitudes. Since competency is observable, it can be measured and assessed to ensure acquisition [10]. Competency based education refers to an approach to preparing health professionals for practice that is fundamentally oriented to graduate outcome abilities and organized around competencies derived from an analysis of societal and patient needs. It involves moving away from a strictly time-based training model towards one that identifies the specific knowledge, skills, and abilities needed for practice [11]. In Canada, the competency based framework for medical education is known as CanMEDS. The CanMEDS framework of physician competencies is used by the Royal College of Physicians and Surgeons for Canadian post-graduate medical training [12]. The CanMEDS framework is organized in seven clusters of competencies, or CanMEDS roles: Medical Expert, Manager, Collaborator, Health Advocate, Communicator, Scholar, and Professional. QI competencies are covered in the CanMEDS Manager role, which empowers physicians to act as integral participants in healthcare organizations, to organize sustainable practices, to make decisions about allocating resources, and to contribute to the effectiveness of the healthcare system. In fact, QI competencies span beyond the Manager role and involves effective integration of the other CanMEDS roles [10].

While many residency training programs have begun to recognize the importance of teaching QI to residents, there is no standardized competency based curriculum on QI that can be readily implemented in residency programs. Currently there is no prospective data on the impact and sustainability of implementing a QI curriculum during internal medicine residency training. It remains unclear whether a competency based QI curriculum is associated with a favourable and sustainable impact in residency training.

Our primary objective was to measure the impact of a novel, competency based curriculum on QI that utilizes both didactic teaching and an experiential longitudinal project in delivering the QI curriculum.

## Methods

### Curriculum content development

The development and implementation of the competency based curriculum on QI has been previously described in detail [13]. Briefly, a comprehensive needs assessment of perceived and non-perceived needs was conducted with stakeholder input from the University of British Columbia (UBC) Internal Medicine residency executive committee, the available literature, local hospitals and health authority regions. Educational objectives based on the CanMEDS [12] framework were then created and incorporated into a two-phase curriculum that was approved by the UBC Internal Medicine residency training committee. Phase 1 consisted of an academic half-day (AHD) didactic curriculum spread over two regularly-scheduled academic half-days, four weeks apart. Phase 2 comprised of a team-based longitudinal QI project spread over twelve months. All first-year residents formed teams of four or five people and selected a project of their own interest while being supervised by faculty mentors with QI experience. To help resident teams achieve important milestones in their QI projects, a total of 5 one-hour longitudinal tutorial sessions were held during the twelve-month project period to provide feedback to each team regarding specific operational issues during the QI process.

At the end of the longitudinal QI project, each resident team produced a QI abstract and presented their projects at a year-end QI Project Podium Presentation Day that was video-conferenced to audiences distributed throughout the province of British Columbia [14]. An invited guest speaker with expertise in QI started the QI day with a keynote presentation on a relevant QI topic, followed by resident team presentations. Each presentation was followed by ten minutes of questions by the audience. The practical aspects of implementing this competency based curriculum on QI have been previously described [15].

We obtained approval from the UBC Internal Medicine residency training committee and obtained ethics approval from the University of British Columbia Behavioural Research Ethics Board. Written informed consent was obtained from all participants.

### Evaluation of the impact of the competency based curriculum on QI

To determine the impact of the QI curriculum, we used standardized and validated tools. The study endpoints were categorized as self-assessment, objective assessment, and performance based assessment.

### Self-assessment

Self-assessment of perceived QI skills was assessed using a standardized, 12-item self-assessment tool previously validated with internal medicine residents [9]. For each item, residents rated their responses on a Likert scale from 1 to 4, with 4 = Extremely comfortable; 3 = Moderately comfortable; 2 = Slightly comfortable; 1 = Not at all comfortable. The questionnaire was administered to all entry level or postgraduate year one (PGY-1) residents at three time points: pre-curriculum, immediately after delivery of the AHD portion of the curriculum (post-curriculum), and twelve months later with completion of the team-based QI projects by the residents (post-project curriculum). The number of residents who gave each rating for each item was recorded. In order to capture self-assessment group data, a weighted mean expressed in percent, known as the satisfaction index, was calculated for each evaluation item [16]. The satisfaction index was used as it was also used by the postgraduate medical education accreditation body (Royal College of Physicians and Surgeons Canada). For example, if thirty residents responded to a particular item, and if two residents gave a rating of 2, ten gave a rating of 3, and eighteen gave a rating of 4, then an overall score of 106 would be obtained (2 × 2 + 10 × 3 + 18 × 4 = 106). The satisfaction index was calculated by multiplying the overall score by a factor of 25 (100 per cent divided by the maximum rating value of 4), and dividing it by the number of participants (thirty). This produced a satisfaction index of 88.3 per cent for this example (106 × 25/30). In general, items with a satisfaction index below 60 per cent may indicate an area of concern, and are worth investigating. An overall satisfaction index for each time point was then calculated by taking the mean of the satisfaction indices for all 12 evaluation items at that time point.

### Objective assessment

We used the Quality Improvement Knowledge Assessment Tool (QIKAT), a standardized and validated method for measuring QI knowledge in internal medicine residents [9]. In this tool, residents responded to three different clinical scenarios and formulate aim statements, QI measures, and possible changes for improvement. Each response was scored based on a standardized answer key that looked at whether the responses incorporated QI fundamentals (e.g. process knowledge) and whether the three elements (aim, measure and change) were clearly related. The answer key came with a Likert scale from 0 (no response) to 5 (excellent; no modifications needed; elements clearly related) that was used to score the answers. The QIKAT was available in two versions: pre-test and post-test, that included different clinical scenarios. The pre-test version was administered to all PGY-1 residents prior to implementation of the curriculum (pre-curriculum), whereas the post-test version was administered post-curriculum. The pre-test version was re-administered twelve months later post-project curriculum. It was felt that the time lapse between pre-curriculum and post-project curriculum was sufficient for adequate washout of any possible learning effect from re-using the same pre-test. An initial training period occurred to ensure consistency of results by the assessor. Specifically, QIKAT scores from a previous year (not included in the present study) were rated by two reviewers independently, and disagreements were resolved by consensus. One assessor then rated resident performance on the objective assessments using the rating scale described above.

### Performance-based assessment

All residents were required to participate in and carry out a QI project. The successful completion and presentation of the project was felt to be an important milestone to demonstrate educational significance, as knowledge and skills learned in QI sessions were applied to a real-life scenario. Projects were evaluated at the annual Resident QI Project Podium Presentation Day based on criteria such as patient focus, knowledge of the QI process, measurements and changes achieved amongst others [14]. Projects were assessed independently by 3 individuals with QI expertise, and a different set of 3 assessors were used each year. For this study we report the titles of these QI projects.

### Data analysis

Each resident was coded with a unique identifier to ensure anonymity to the investigators and data analysts. A database of performance on each of the items of the self-assessment and objective assessment questionnaires was created using the anonymous resident data.

The distributions of demographics, specifically academic year, sex, and whether the resident had participated in quality improvement previously, were described using frequencies and percentages. To compare the distribution of categorical variables across the pre-curriculum, post-curriculum and post-project curriculum time points, we used the Friedman test, which accounted for the multiple measurements from a resident. This test yielded a chi-square statistic and p value as a measure of difference in distributions over time. To compare the average self-assessment and objective assessment scores over time, we used the repeated measures ANOVA. This yielded an F-statistic and p value as a measure of difference in averages over time. Paired t-tests were used to assess the impact of the curriculum at the three time points: a) post-curriculum to pre-curriculum; b) post-project curriculum to pre-curriculum and c) post-project curriculum to post-curriculum. Group means and individual mean change scores were assessed.

## Results

A total of 175 PGY-1 internal medicine residents over four academic years (2007 – 2011) participated in the study with baseline demographics shown in Table 1. A response rate of 160/175 (91%) was seen at post-curriculum, and 114/175 (65%) post-project curriculum. The majority of residents (86.9%, n = 152) at baseline had not participated in any quality improvement previously. Yet, when asked whether they felt confident in making a change to improve health, 74% felt “reasonably confident” or “confident”, while 23% of residents felt “not confident”. As the curriculum continued, the proportion of residents’ reporting being “confident” or “reasonably confident” increased to 86.6% post-curriculum, and at twelve months post-project curriculum this confidence was sustained in 89.3% of residents (Table 2).

**Table 1:** **Demographics of 175 PGY1 residents from 2007/2008 to 2010/2011**

**Characteristic**	**Pre-curriculum**		**Post-curriculum**		**Post-project curriculum**	
	n*	**(%)**	**n** ^**†**^	**(%)**	**n** ^**‡**^	**(%)**
**Academic year**						
2007/2008	36	(20.6)	34	(21.3)	31	(27.2)
2008/2009	48	(27.4)	45	(28.1)	30	(26.3)
2009/2010	49	(28.0)	45	(28.1)	27	(23.7)
2010/2011	42	(24.0)	36	(22.5)	26	(22.8)
**Sex**						
Male	102	(58.3)	89	(55.6)	62	(54.4)
Female	73	(41.7)	71	(44.4)	52	(45.6)
**Participated in QI previously**						
Yes	23	(13.1)				
No	152	(86.9)				

Abbreviation: QI = quality improvement.

*4 residents with data not available at pre-curriculum.

^†^19 residents with data not available at post-curriculum.

^‡^65 residents with data not available at post-project curriculum.

**Table 2:** Confidence in making change to improve health among 175 residents from 2007/2008 to 2010/2011*

**Confidence in making change to improve health care**	**Pre-curriculum**		**Post-curriculum**		**Post-project curriculum**	
	**n** ^**†**^	**(%)**	**n** ^**‡**^	**(%)**	**n** ^**§**^	**(%)**
Not confident	40	(23.0)	21	(13.4)	12	(10.7)
Reasonably confident	86	(49.4)	90	(57.3)	63	(56.3)
Confident	48	(27.6)	46	(29.3)	37	(33.0)

*Distribution of confidence was statistically different across time (chi-square statistics = 12.1, degrees of freedom (df) = 2, p = 0.002).

^†^5 residents with data not available at pre-curriculum.

^‡^22 residents with data not available at post-curriculum.

^§^67 residents with data not available at post-project curriculum.

### Self-assessment

**Figure 1:**
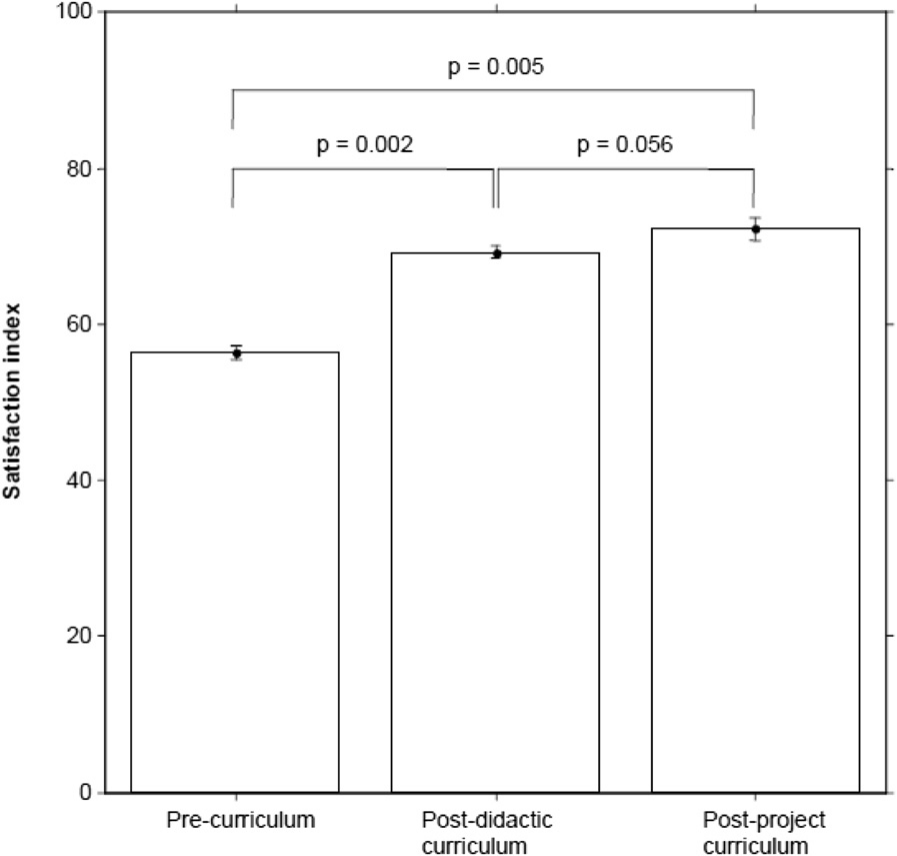
**Impact of curriculum on self-assessment of QI skills in as measured by average difference in satisfaction index.**

Self-assessment of QI skills improved from baseline as determined by the average difference in satisfaction index (group data) as well as in individual self-assessment scores. At the pre-curriculum stage, the satisfaction index increased from 56.4% to 69.2% at the post-curriculum stage (p-value: 0.002), and to 72.2% at post-project curriculum (p-value: 0.005 from baseline), as demonstrated in Figure 1. When reviewing individual self-assessment scores, the effect of the curriculum also showed statistically significant improvements from baseline compared to all other time points, although for some items (e.g. applying statistical process control) the change from post-curriculum to post-project curriculum was not significant (Table 3).

**Table 3:** **Impact of curriculum on self-assessment of QI skills as measured by average difference in individual scores**

**Time**	**Average individual score**	**SD of individual score**	**Difference from pre-curriculum (95% CI)**	**p value for difference from pre-curriculum**
Q1: Defining a clear problem statement (goal, aim)*				
Pre-curriculum	2.6	0.7	–	–
Post-curriculum	3.0	0.5	0.4 (0.26, 0.57)	<0.001
Post-project curriculum	3.1	0.5	0.5 (0.33, 0.64)	<0.001
**Q2: Applying best professional knowledge** ^**†**^				
Pre-curriculum	2.4	0.7	–	–
Post-curriculum	2.8	0.6	0.4 (0.27, 0.56)	<0.001
Post-project curriculum	3.1	0.6	0.7 (0.53, 0.86)	<0.001
**Q3: Developing appropriate measures** ^**‡**^				
Pre-curriculum	2.2	0.7	–	–
Post-curriculum	2.8	0.6	0.6 (0.43, 0.76)	<0.001
Post-project curriculum	2.9	0.6	0.7 (0.56, 0.87)	<0.001
**Q4: Studying the process of care** ^**§**^				
Pre-curriculum	2.2	0.7	–	–
Post-curriculum	2.7	0.6	0.5 (0.40, 0.73)	<0.001
Post-project curriculum	2.8	0.6	0.6 (0.47, 0.76)	<0.001
**Q5: Developing a data collection plan consistent with time and resource limitations** ^**||**^				
Pre-curriculum	2.2	0.7	–	–
Post-curriculum	2.8	0.6	0.6 (0.47, 0.80)	<0.001
Post-project curriculum	3.0	0.6	0.8 (0.71, 1.02)	<0.001
**Q6: Analyzing data** ^**¶**^				
Pre-curriculum	2.3	0.8	–	–
Post-curriculum	2.7	0.7	0.4 (0.21, 0.53)	<0.001
Post-project curriculum	2.7	0.7	0.4 (0.18, 0.57)	<0.001
Q7: Applying statistical process control**				
Pre-curriculum	1.9	0.8	–	–
Post-curriculum	2.5	0.9	0.6 (0.43, 0.76)	<0.001
Post-project curriculum	2.5	0.7	0.6 (0.40, 0.72)	<0.001
**Q8: Describing the roles of different professionals in health care improvement** ^**††**^				
Pre-curriculum	2.5	0.7	–	–
Post-curriculum	2.9	0.6	0.4 (0.33, 0.62)	<0.001
Post-project curriculum	3.1	0.6	0.6 (0.50, 0.81)	<0.001
**Q9: Implementing a structured plan to test a change** ^**‡‡**^				
Pre-curriculum	2.2	0.7	–	–
Post-curriculum	2.9	0.6	0.6 (0.45, 0.76)	<0.001
Post-project curriculum	3.0	0.6	0.8 (0.61, 0.94)	<0.001
**Q10: Sustaining a change over time** ^**§§**^				
Pre-curriculum	2.2	0.7	–	–
Post-curriculum	2.6	0.7	0.4 (0.20, 0.52)	<0.001
Post-project curriculum	2.7	0.6	0.5 (0.33, 0.67)	<0.001
**Average of Q1–Q10** ^**||||**^				
Pre-curriculum	2.3	0.5	–	–
Post-curriculum	2.8	0.5	0.5 (0.40, 0.61)	<0.001
Post-project curriculum	2.9	0.4	0.6 (0.51, 0.73)	<0.001

Abbreviations: SD = standard deviation; CI = confidence interval.

*There was a difference in average individual scores across time (F-statistic = 19.9; df = 2, 104; p <0.001).

^†^There was a difference in average individual scores across time (F-statistic = 35.5; df = 2, 104; p <0.001).

^‡^There was a difference in average individual scores across time (F-statistic = 44.7; df = 2, 104; p <0.001).

^§^There was a difference in average individual scores across time (F-statistic = 37.7; df = 2, 104; p <0.001).

^||^There was a difference in average individual scores across time (F-statistic = 60.9; df = 2, 104; p <0.001).

^¶^There was a difference in average individual scores across time (F-statistic = 12.0; df = 2, 104; p <0.001).

**There was a difference in average individual scores across time (F-statistic = 32.2; df = 2, 104; p <0.001).

^††^There was a difference in average individual scores across time (F-statistic = 36.6; df = 2, 104; p <0.001).

^‡‡^There was a difference in average individual scores across time (F-statistic = 48.0; df = 2, 104; p <0.001).

^§§^There was a difference in average individual scores across time (F-statistic = 17.6; df = 2, 104; p <0.001).

^||||^There was a difference in average individual scores across time (F-statistic = 68.6; df = 2, 104; p <0.001).

### Objective assessment

**Figure 2:**
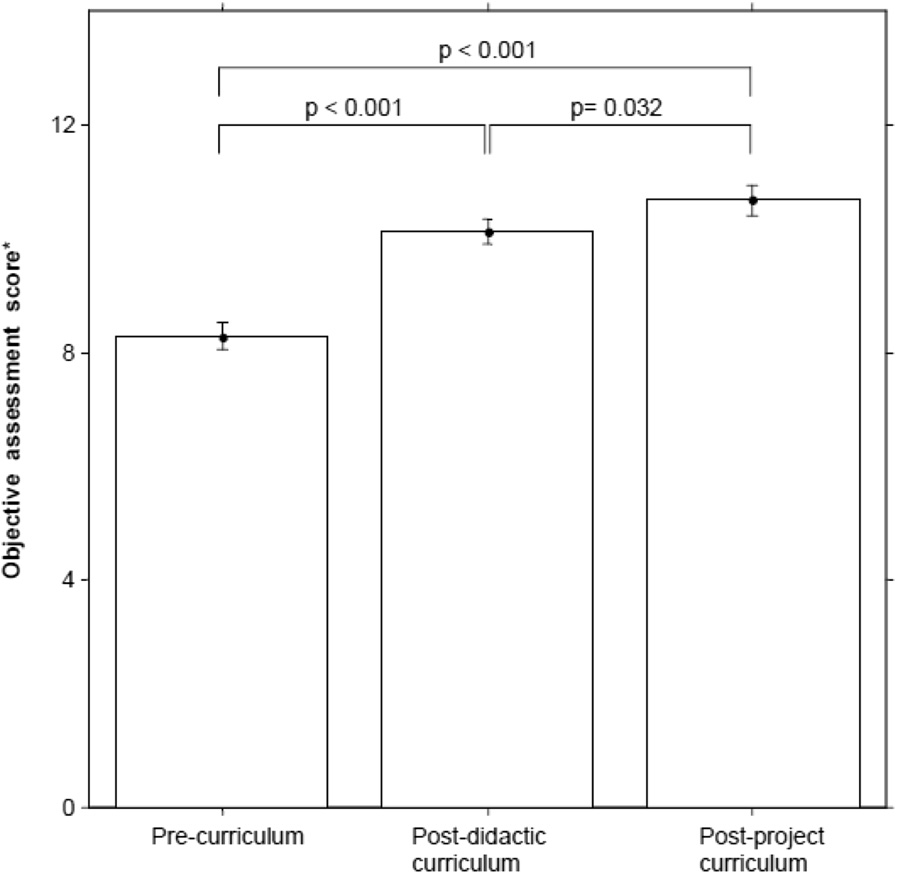
**Impact of curriculum on objective assessment of QI knowledge in 2007/08–2010/11, as measured by average difference in individual scores.** *There was a difference in average individual scores across time (F-statistic = 51.2; df = 2, 104; p < 0.001).

Objective assessment of QI knowledge using the QIKAT found that average individual scores increased significantly from 8.3 out of 15 (Standard Deviation [SD] 2.5) at pre-curriculum to 10.1 out of 15 (SD 2.3) post-curriculum (difference from pre-curriculum 1.9, 95% Confidence Interval [CI] (1.39, 2.32); p-value: <0.001). At post-project curriculum, QIKAT scores improved further to 10.7 (difference from pre-curriculum 2.4, 95% CI (1.89, 2.91); p-value: <0.001 from baseline). The improvement from post-curriculum to post-project curriculum was also statistically significant (difference from post-curriculum 0.5, 95% CI (0.05, 1.04); p-value: 0.032), as seen in Figure 2.

### Performance-based assessment

Over the course of four academic years, multiple QI projects were presented at an annual resident QI day. All QI projects were successfully executed over the years (100% implementation). Table 4 highlights the scope and breadth of projects undertaken by the residents, with some being published or presented at various other conferences. When we discussed with the residents what they learned from the QI projects, they most commonly reported a sense of empowerment to “make a difference” when they encountered an improvement opportunity clinically. A small number of residents had moved on to pursuing special training in QI after exposure to this curriculum, and some had made QI their academic career trajectory.

**Table 4:** **QI projects completed by Internal Medicine Residents**

**Year**	**Resident QI project title**
**2007**	Effect of implementing a standardized electronic order set for admission of septic patients to the Clinical Teaching Unit (CTU) on rates of mortality and Intensive Care Unit (ICU) transfers
	Intravenous (IV) Antibiotic auto-stop: good or bad? Evaluation of the St. Paul’s Hospital (SPH) 3-day IV antibiotic automatic stop date
	Evaluating the uptake of delirium pre-printed orders to improve recognition and management of delirium in hospitalized older adults
	Transfusion Medicine: improving turnaround time for packed red blood cell transfusions
	Effect of an information pamphlet with short introduction on management of hypokalemia in patients admitted to a medical CTU
	The effect of an educational session outlining indications for pneumococcal vaccination on rates of vaccination assessment at the time of admission to a clinical teaching unit at a tertiary care hospital
	Daily weights for patients with chronic heart failure: weighing in on strategies to improve their completion
**2008**	Mock codes in the ICU: effect on perceived confidence and comfort levels in code blue situations
	Decreasing rate of error in ultrasound guided taps: DARE-US Study
	Documenting code status at the time of admission to hospital
	The effect of a contrast induced nephropathy flowsheet on practice variability at St. Paul’s Hospital in-patients
	A Quality Improvement project to reduce the indwelling time of central venous catheters in ICU patients transferred to medical wards
	Order entry sets implemented for Vancouver General Hospital CTU to improve completeness and appropriateness of cerebrospinal fluid testing (CSF) for meningitis
	Stop the Clot - Decreasing length of time to achieving therapeutic INR in patients with deep vein thrombosis (DVT), pulmonary embolism (PE), and atrial fibrillation: the impact of the introduction of a nomogram protocol for warfarin dosing
**2009**	Improving thromboprophylaxis in medical inpatients
	Decreasing contamination of stethoscopes by potentially pathogenic microorganisms
	Reduction of daily bloodwork through modified ordering practices on clinical teaching units
	Improving pneumococcal vaccination rates in patients with chronic obstructive pulmonary disease
	Inappropriate foley catheter use on the general medicine ward: a quality improvement initiative to investigate the scope and reduce the burden of the problem
	Reducing time to IV antibiotic delivery in CTU patients
	A quality improvement project designed to encourage elevation of the head of bed in ventilated, critical care patients at Vancouver General Hospital
	Increasing adherence to guidelines on the use of proton pump inhibitors for gastroprotection among aspirin users with high risk features for upper gastrointestinal complications
**2010**	A Vision for Quality Improvement: increasing the frequency of calls to the British Columbia Eye Bank from CTU wards
	Calcium and vitamin D supplementation: increasing utilization in those at risk for osteoporosis
	Improving nicotine replacement therapy utilization using a CTU preprinted order set at Royal Columbian Hospital
	Increasing the use of bowel protocol in CTU patients through stamp orders
	Electronic medication reconciliation through use of preprinted transfer orders in the ICU
	SBAR (Situation, Background, Assessment, Recommendation): Introduction of SBAR in the resident way of thinking and communication
	Improvement on pulmonary rehabilitation referral after hospitalisation for acute exacerbation of chronic obstructive pulmonary disease (COPD): a quality improvement initiative
	Novel goals of care documentation for Vancouver General Hospital CTU
	Management of hyperkalemia in hospitalized CTU patients
	Vaccination for Hepatitis A in patients with chronic liver diseases

## Discussion

Acquisition, application and sustainability of quality improvement skills are considered to be core competencies by the Royal College of Physicians and Surgeons Canada under the CanMEDS Manager role. Accordingly, we developed a competency based curriculum on QI using the CanMEDS framework for internal medicine residents and demonstrated that it may help to developing residents’ QI knowledge and skills during their residency. Residents’ QI knowledge improved after didactic curriculum. This improvement was sustained at twelve months post-project curriculum with the completion of the residents’ team-based QI projects, suggesting experiential learning may be a helpful component of maintaining knowledge.

The main strength of this study was that QI knowledge (measured and self-reported) could improve with didactic teaching alone. This is important since many of the barriers and challenges encountered relate to the actual QI project. A corollary for medical educators is that if the main objective is to improve knowledge of QI, this could be achieved with didactic teaching on QI. Another main strength of this study was that results were sustained at 12 months, and engagement in experiential learning may importantly contribute to such sustainability of QI knowledge (as shown by the statistically significant increase in QIKAT scores comparing post-curriculum assessment and post-project assessment). Importantly, resident self-reported confidence in making changes to improve health care was also sustained at one year after completion of the QI project.

We reported residents’ self-assessment scores (Table 3) using mean scores as the same reporting method was used in the literature [9]. We felt reasonably comfortable that the statistically significant improvement in mean scores that we observed in our study were within the same order of magnitude as the improved scores reported in the literature [9], therefore at a minimum we might conclude that we observed a similar degree of clinical improvement in residents’ self-assessment scores as reported in the literature. We understand that the exact degree to which these self-reported measures correlated with actual knowledge/skills requires further study. We therefore decided to complement the self-assessment measures with the objective knowledge scores (using the QIKAT). The QIKAT was previously validated and its correlation with clinical improvement previously reported [9]. We observed similar improvement in the objective knowledge scores among our resident participants, and the improvement was sustained over the 12-month period during which the longitudinal QI projects were implemented by the residents. Moreover, the improvement in QIKAT scores in our study was in a similar order of magnitude as reported in the literature [9]. Again at a minimum we can conclude that our study findings confirm the literature.

Other studies in the literature have found transient improvements in QI knowledge, often after a short period of time. One example involved creating an interdisciplinary four-week QI elective rotation involving 5 residents (from family medicine and internal medicine) and 2 master’s-level nursing students. Using a pre-post comparison, the authors demonstrated statistically significant improvement in QIKAT scores after working on a QI project [17]. Another study examined 11 internal medicine residents in a 4-week practice-based learning and improvement (PBLI) elective that utilized didactic and experiential learning through projects that were completed within the 4-week elective [9]. PLBI is a competency of the Accreditation Council for Graduate Medical Education for lifelong learning and quality improvement [18]. Objective measures using the QIKAT tool found statistically significant improvements in participants compared to a control group after the elective. At six months, self-assessed proficiency in PBLI was maintained [9]. In another study, 44 internal medicine and pediatrics residents participated in a one-month continuous quality improvement (CQI) curriculum within an ambulatory rotation block. The curriculum, which comprised of three 1-hour portions - a didactic session describing QI theory, a “brainstorming” session for potential projects and a presentation session where residents presented their project to the resident group, found that the CQI curriculum significantly improved residents’ knowledge, perceived knowledge and self-efficacy [8].

Our study involved a larger number of internal medicine residents over several years (albeit numbers still limited to a single institution), and we had residents complete a longitudinal project over twelve months that allowed residents to apply QI principles through several Plan-Do-Study-Act cycles, and measuring QI knowledge at the completion of their QI projects to assess retention of QI knowledge. The exact mechanisms of how QI knowledge could be sustained by participating in project-based experiential learning remains unclear. The potential role of experiential project work in competency based QI curriculum for sustainable impact has been reported by others as well [9,19,20].

We observed that the scope of the QI projects continued to broaden over the years. One possible explanation was that there might be some degree of “meta-learning” among the resident classes as the senior residents became more comfortable and/or engaged in QI (that is, this might signify the beginning of an underlying cultural shift in favour of QI, although we did not study this outcome specifically in this study). Some of the more common barriers encountered in developing the QI projects included the residents’ difficulty in selecting a single QI project to focus on because they had numerous interesting ideas, identifying a faculty sponsor with QI expertise (although many had clinical content expertise), narrowing the scope of the project (residents were constantly reminded that they could not “change the world” with a single QI project), engaging QI analysts in the health organisations to assist in the QI projects, and presenting data in run charts/control charts format, to name a few. We also observed that all QI projects selected by residents were based within a hospital setting, and none were ambulatory in nature despite repeated encouragement to conduct a project in the outpatient setting. Further study as to the reasons why this occurred are worth exploring.

Despite the above, we did encounter several barriers in this study. To implement the curriculum, we continued to face the challenge of identifying adequate infrastructure, dedicated teaching time, and willing teachers. Furthermore, the project-based portion of the QI curriculum required hospital staff with appropriate projects that could be finished within a twelve-month period, while implementation of these projects required hospital and institutional “buy-in” to support and allow projects to go ahead. Lastly, residents anecdotally reported frustration with balancing clinical workload and an additional QI project amongst other research interests. However, resident concerns were counter-balanced by feelings of satisfaction and empowerment to utilize QI principles when faced with system inefficiencies clinically. These learner, teacher, curricular and environmental factors are important considerations for successful implementation of a QI curriculum [21].

There were several other limitations. As the curriculum progressed, attendance at AHD by residents decreased with time, resulting in high attrition that reflects a common challenge when delivering educational program in a busy residency. Possible reasons include competing interests of heavy clinical workload during residency and/or possibly decay in interest in QI – our study was not designed to identify a single root cause. We recognize that self-assessment can be subjective and introduce bias, but we tried to minimize this by anonymizing all resident responses. The QIKAT, while validated and shown to have good construct and predictive validity, has been criticized by some as having poor reliability. However, at the time of study there were no other assessment tools available to evaluate QI learning. Future studies may wish to utilize newer tools such as the QIKAT-Revised [22], which was recently shown to increase inter-rater reliability. The performance-based assessment here was also deliberately generalized. This was a single institution study, thus potentially limiting generalizability to other programs, settings or countries. It should be noted, however, that our curriculum has been replicated and implemented in other Canadian Faculties of Medicine, suggesting it could be generalizable. In doing so, mitigation strategies should be developed to overcome the difficulty in recruiting teachers over time, and to sustain the interest of residents in the midst of busy residency programs.

## Conclusion

We developed a novel competency based curriculum on QI for internal medicine residents and demonstrated improvement in QI knowledge (self-assessment and objective assessment) with didactic curriculum, and such knowledge improvement was sustained over a twelve-month period post-project curriculum. Future studies should be directed at the feasibility of incorporating QI curricula into other specialty programs as well as the impact of QI curricula on improving patient outcomes.
